# Proton-Sensing G Protein-Coupled Receptors and Their Potential Role in Exercise Regulation of Arterial Function

**DOI:** 10.3390/biom15060813

**Published:** 2025-06-04

**Authors:** Fengzhi Yu, Dandan Jia, Ru Wang

**Affiliations:** School of Exercise and Health, Shanghai University of Sport, Shanghai 200438, China; 2311516003@sus.edu.cn

**Keywords:** exercise, proton-sensing G protein-coupled receptors, vascular endothelial cell, vascular smooth muscle cell, arterial function

## Abstract

During periods of exercise, the primary cause of metabolic acidosis is the accumulation of lactate from anaerobic metabolism, whereas a transient increase in CO_2_ triggers a mild respiratory acidosis through the production of carbonic acid (H_2_CO_3_). The combined effects of these reactions result in a slight acidifying shift in arterial blood pH. Proton-sensing G protein-coupled receptors (including GPR68, GPR4, GPR132, and GPR65) represent the primary receptors within the body for detecting alterations in extracellular proton concentrations. These receptors have been demonstrated to possess potential roles in mechanosensation, intestinal inflammation, oncoimmunological interactions, hematopoiesis, as well as inflammatory and neuropathic pain. Recent studies have shown that the activation or inhibition of these receptors modulates a number of arterial functions, including angiogenesis, arterial relaxation, and arterial inflammation. It is well established that moderate exercise has a beneficial effect on the regulation of arterial function. This study examines the effect of exercise on proton concentrations in the microenvironment of the organism and its influence on proton-sensing G protein-coupled receptors located on cell membranes, as well as possible mechanisms involved in the regulation of arterial function. The objective is to present novel perspectives for the exploration of potential drug targets for the prevention and treatment of arterial dysfunction and the development of exercise regimens.

## 1. Introduction

Cardiovascular diseases (CVDs) are responsible for more than 17 million disease-related deaths worldwide each year, representing the leading cause of death [1,2]. Approximately 35.6 million deaths will be attributed to CVDs by 2050 [3]. Atherosclerosis represents a fundamental pathological marker for the assessment of cardiovascular well-being and is strongly associated with the incidence of CVDs and all-cause mortality. Increased arterial stiffness, which is primarily caused by structural abnormalities of the arterial wall [4], can result in an elevated left ventricular afterload through the action of artery–left ventricular coupling mechanisms. This may result in additional damage to other organs, including the heart and brain [5]. Impaired endothelium-dependent vasodilator function is regarded as a pivotal instigator of atherosclerosis [6,7]. There is a growing awareness of the potential benefits of enhancing arterial diastolic function in reducing the morbidity and mortality associated with CVD.

A substantial body of evidence has accumulated over the years, establishing the efficacy of exercise in the prevention and treatment of CVDs [8,9]. The current mechanisms by which exercise affects arterial function are largely manifested through the regulation of vascular endothelial cell (VEC) and vascular smooth muscle cell (VSMC) activity. This includes the regulation of endothelial cell integrity, which results in the improvement of selective barrier function [10], reduced adhesion to leukocytes [11], anti-thrombogenicity [12], angiogenesis, and regulation of vascular tone [13]. It also encompasses the modulation of endothelial function, including the regulation of increased flow shear stress [14], and the attenuation of inflammatory processes [15,16]. Additionally, it has been demonstrated that exercise may have a beneficial effect on atherosclerosis, including increased collagen content and decreased intercellular adhesion molecule-1 [17]. These adaptations have been associated with a reduction in both necrotic core area and plaque burden [18]. Furthermore, structural adaptations have been observed following exercise, including improvements in coronary artery size [19] and distensibility [20], increases in lumen diameter [21], and reductions in wall thickness [22].

During exercise, the body’s metabolic rate increases to meet the energy demands of working muscles, leading to enhanced production of CO_2_. The rise in CO_2_ promotes the formation of H_2_CO_3_, contributing to a mild respiratory acidosis. However, this effect is largely counteracted by compensatory hyperventilation, which rapidly eliminates excess CO_2_. The predominant cause of acidification in intense exercise is instead lactic acid accumulation from anaerobic metabolism, driving metabolic acidosis and a slight decrease in arterial blood pH [23,24]. VECs and VSMCs within arteries express proton-sensing G protein-coupled receptors (GPCRs), including the ovarian cancer G protein-coupled receptor (OGR1) (also known as GPR68), GPR4, G2 accumulation protein (G2A) (also known as GPR132), and T-cell death-associated gene 8 (TDAG8) (also known as GPR65) [25,26,27,28,29]. Such receptors are capable of detecting alterations in extracellular pH levels, which then trigger intracellular signaling cascades [30,31]. These receptors are expressed in diverse cells that regulate central pH homeostasis, pH sensing in the immune system, and vascular responses to pH [32]. In the cardiovascular system, the binding of excess protons in the acidic extracellular medium to receptors on the cell membrane results in alterations to receptor shape and the subsequent activation or inhibition of downstream signaling in VECs and VSMCs [33,34]. These interactions then regulate a number of processes, including angiogenesis, arterial diastole, and inflammation. The objective of this review was to examine the effect of physical activity on the concentration of protons in the microenvironment within the organism and to analyze whether these protons can act via proton-sensing GPCRs on the membranes of VECs and VSMCs, leading to improved arterial function.

## 2. Overview of Proton-Sensing GPCRs

### 2.1. Structure and Function

GPCRs, also known as 7-transmembrane structural domain receptors (7TMRs), constitute a diverse family of proteins that are encoded by the human genome. To date, more than 800 GPCRs have been identified [35]. The intracellular pH of cells is regulated within the range of 7.1 to 7.2 by the control of membrane proton pumps and transport proteins, through the utilization of pH sensors located in the cell membrane. Alterations in extracellular pH changes lead to the activation of specific acid-sensitive ion channels (ASICs), proton-sensing GPCRs, and lactate receptors, among others, which regulate cellular function [36]. Proton-sensing GPCRs are capable of detecting alterations in the concentration of H^+^ in the vicinity of histidine residues that are present in their extracellular domains.

Proton-sensing GPCRs are involved in a number of biological processes, including cell proliferation, metastasis, angiogenesis, apoptosis, immune cell functioning, and inflammatory responses [37,38]. Members of the pH-sensing GPCR family, including GPR68, GPR4, GPR132, and GPR65, have the capacity to detect alterations in extracellular proton concentrations [39] (see Table 1). The receptors demonstrate minimal activity at pH 7.6–7.8 and attain full activation at the slightly lower pH range of 6.4–6.8 [40]. In physiological conditions, GPR4, GPR68, and GPR132 are expressed in a ubiquitous manner. However, GPR132 shows the strongest expression in leukocytes, including T- and B-cells, neutrophils, and macrophages. In contrast, GPR65 is expressed almost exclusively in lymphoid tissue [41]. With regard to the context of arteries, proton-sensing GPCRs are predominantly expressed in VECs and VSMCs.

### 2.2. Signaling Pathway

GPCRs are capable of transducing signals from the external environment by activating G proteins, including Gαi/o, Gαq/11, Gαs, and G12/13. These proteins regulate the activity of specific enzymes, ion channels, and proteins involved in signaling cascades [42]. It is worth noting that proton-sensing GPCRs do not bind complex extracellular ligands; however, they are involved in heterotrimeric G protein responses when the extracellular proton concentration increases slightly [43]. In the presence of an extracellular acidic microenvironment, these receptors become active, and their activation of multiple downstream pathways plays a crucial role in cellular functions and processes.

Several models of the mechanism by which proton-sensing GPCRs recognize protons have been proposed. Proton-sensing GPCRs harbor an abundance of extracellular histidine residues that likely titrate at physiologically relevant pH levels [44]. The prevailing perspective suggests that proton-sensing GPCRs detect the acidic extracellular environment through a process involving protonation and coordination of several amino acids, including extracellular histidines and buried amino acid triads containing aspartic acid and two glutamic acids [43,44,45]. Subsequent endocytosis of proton-sensing GPCRs transports them to the acidic endosomal compartment, where there is a greater likelihood of receptor activation [45]. However, mutational studies suggest that histidines are dispensable for proton sensing, as the removal of all extracellular histidines does not abolish proton-driven activation in GPR68 [32,46]. At this juncture, the mechanisms by which protons activate proton-sensing GPCRs, whether proton-sensing GPCRs can be selectively activated in endosomal compartments, and whether they follow the tight coupling of transport and signaling described for most known GPCRs, remain to be elucidated.

**Figure 1:**
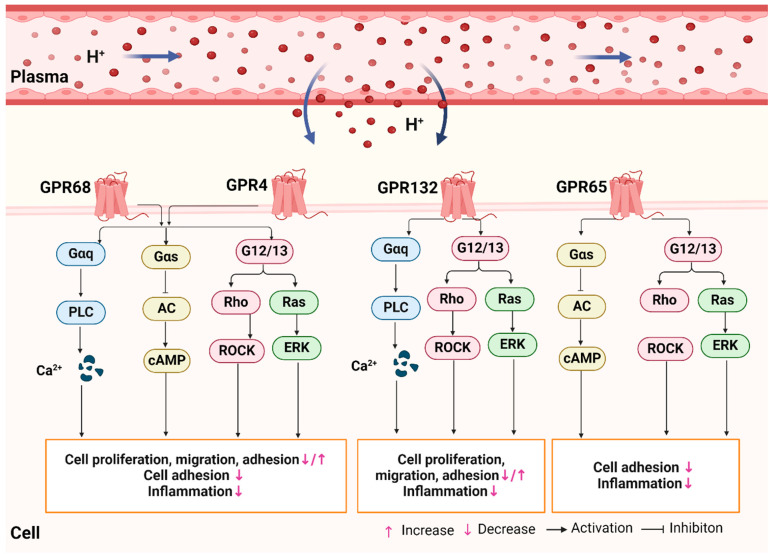
Proton-sensing GPCR-related signaling pathway. A reduction in extracellular pH enables the activation of GPR68, GPR4, GPR65, and GPR132 receptors located on the cell membrane. The coupling of Gαq proteins can inhibit cell proliferation, migration, and adhesion. The coupling of Gαs proteins has been demonstrated to reduce cell adhesion and inflammation levels. Furthermore, Gαs proteins play a role in inflammatory regulation, with the activation of G12/13 and the inhibition of pro-inflammatory cytokine secretion. This figure was created using BioRender. Abbreviations: PLC: phospholipase C; AC: adenylate cyclase; cAMP: cyclic adenosine monophosphate; Rho: Ras homologous; ROCK: Rho-associated protein kinase; Ras: Rat sarcoma; ERK: extracellular signal-regulated kinase; PKA: protein kinase A; NF-κB: nuclear factor kappa-B.

Existing studies have found that GPR68 displays weak activity at pH 7.8 but is fully active at pH 6.8 [44]. In instances of extracellular acidosis, GPR68 may interact with a range of G proteins, thereby initiating signaling cascades. Upon coupling with Gαq/11 protein, it is capable of activating the phospholipase C (PLC)/Ca^2+^ signaling pathway [40]. Activation of the PLC/Ca^2+^ signaling pathway in human ovarian cancer cells has been demonstrated to inhibit cell proliferation and migration. Furthermore, it has been demonstrated to increase the expression of proteins such as extracellular matrix fibronectin, ovalbumin, and collagen type I and type IV, while also enhancing cellular adhesion [47]. Upon coupling with Gαs and G13 proteins, GPR68 can activate the Gαs/cyclic adenosine monophosphate (cAMP) and Ras homologous (Rho)/Rho-associated protein kinase (ROCK) pathways, thereby participating in cell physiological regulatory processes [48]. The coupling of GPR68 with Gαq and Gαs proteins has been demonstrated to regulate cell proliferation, migration, and adhesion [49]. In addition, GPR65 and GPR4 are capable of binding to Gαs proteins, which activates the downstream adenylate cyclase (AC)/cAMP signaling pathway and plays a role in regulating the inflammatory process [50,51]. Additionally, GPR65 has been demonstrated to interact with G12/13 proteins, which may play a role in the activation of the Rho signaling pathway and subsequently inhibit the production of pro-inflammatory cytokines and chemokines [52]. In the event of carbonic acidosis, GPR4 activates the Gαs/cAMP/exchange proteins and Gαq/PLC/Ca^2+^ and G13/Rho signaling pathways, which in turn increases the adhesion of VECs to leukocytes, thereby indirectly exacerbating the inflammatory response [53]. Other studies have demonstrated that GPR132 is responsive to the acidic milieu of plasma, which results in the activation of several downstream conductance pathways, including PLC/Ca^2+^, G13/Rho, and Ras/extracellular signal-regulated kinase (ERK). Furthermore, these pathways are also actively involved in the processes of oxidative stress and inflammation [48,54]. Collectively, proton-sensing GPCRs exert a pivotal influence on a number of cellular processes, including proliferation, migration, adhesion, and inflammatory responses (see Figure 1).

## 3. The Role and Mechanism of Proton-Sensing GPCRs in the Regulation of Arterial Function

Arterial dysfunction is a broad term that encompasses a range of pathological states. These include, but are not limited to, endothelial dysfunction, arterial stiffness, atherosclerosis, hypertension, and abnormal angiogenesis. The regulation of arterial function is primarily mediated by VECs and VSMCs. This section presents a synthesis of existing literature to evaluate the regulatory functions and potential mechanisms of proton-sensing GPCRs in the pathophysiology of arterial function.

### 3.1. GPR68/OGR1

GPR68, also known as OGR1, is located on chromosome 14q31 and serves as a sensor for alterations in extracellular hydrogen ion concentration [25]. It has been demonstrated that the expression of GPR68-dependent genes induced by extracellular acidification is significantly enhanced under hypoxic conditions [55]. GPR68 has been linked to a number of physiological processes, including renal function and bone metabolism [56,57]. A recent observation reported that a homozygous loss of function of GPR68 was described in families with amelogenesis imperfecta, which suggests that GPR68 is required for dental enamel formation [55]. In the arteries, GPR68 is predominantly expressed in endothelial cells of small-diameter vessels [25]. The results of studies conducted on GPR68 knockout mice indicate that GPR68 plays a crucial role in the vasodilation and outward remodeling of small-diameter arteries [25]. Furthermore, GPR68 plays a pivotal role in regulating inflammation, promoting cellular proliferation, facilitating migration and adhesion, as well as promoting angiogenesis [58].

**Figure 2:**
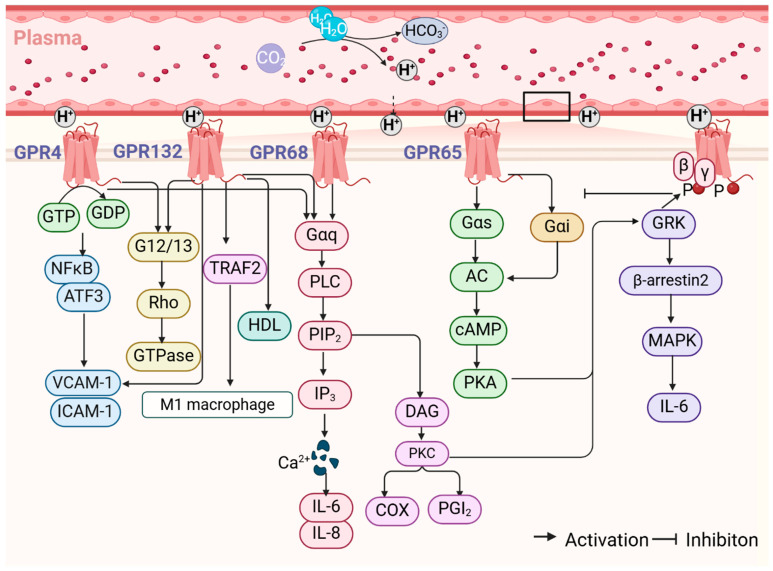
Proton-sensing GPCRs regulate the mechanism related to arterial function. The diffusion of CO_2_ from cellular metabolism into the extracellular fluid and bloodstream generates acidic ions, including H^+^. These ions have the potential to activate proton-sensing GPCRs located on the cell membrane surface. The activation of proton-sensing GPCRs may play a critical role in the regulation of macrophage differentiation, modulation of endothelial dysfunction, neovascularization, and arterial inflammatory responses. This figure was created using BioRender. Abbreviations: NADH: reduced form of nicotinamide-adenine dinucleotide; NAD^+^: nicotinamide adenine dinucleotide; CO_2_: carbon dioxide; H^+^: hydrion; H_2_O: water molecule; HCO_3_^−^: bicarbonate radical; NFκB: Nuclear factor-k-gene binding; ATF3: activating transcription factor 3; VCAM-1: vascular cell adhesion molecule-1; ICAM-1: intercellular adhesion molecule-1; TNF-α: tumor necrosis factor-α; HDL: high-density lipoprotein; GTP: guanosine triphosphate; Rho: Ras homologous; GTPase: guanosine triphosphatase; PLC: phospholipase C; PIP_2_: guanosine triphosphatase; IP_3_: inositol triphosphate; DAG: diacylglycerol; PKC: protein kinase C; COX: cyclooxygenase; PGI_2_: prostaglandin-I-2; GRK: G protein-coupled receptor kinase; AC: adenylate cyclase; cAMP: cyclic adenosine monophosphate; PKA: protein kinase A; MAPK: mitogen-activated protein kinase; IL-6: interleukin-6; IL-8: interleukin-8.

Research addressing the regulation of arterial function by GPR68 has predominantly concentrated on the in vitro cellular level. In an environment with low acidity, GPR68 has the capacity to activate the ERK and mitogen-activated protein kinase (MAPK) signaling pathways, which in turn results in the production of inflammatory factors, such as interleukin-6 (IL-6) [59]. In the presence of an extracellular acidic microenvironment, GPR68 facilitates the formation of basal stress fibers comprising F-actin, thereby enhancing the function of the epithelial barrier [60]. Activation of the PLC/cyclooxygenase (COX)/prostaglandin-I-2 (PGI2) pathway was also observed, resulting in the production of PGI2 and the accumulation of cAMP in aortic smooth muscle cells (AoSMCs) [61]. Liu et al. [62] discovered that the extracellular acidification of the GPR68/Gαq/11 pathway can influence the expression of the COX-2 protein and mRNA in AoSMCs, resulting in the production of PGI2 and cAMP. These ultimately act in concert with lysophosphatidic acid (LPA)/Gαi to elicit an anti-atherosclerotic effect. The results of the cell-based experiments demonstrated that GPR68 activity increased in response to a decrease in the pH of the surrounding environment. Additionally, a medium with a pH of 6.4 was observed to inhibit the proliferation, migration, and tube-forming process of endothelial progenitor cells (EPCs). The effects of this environment on EPCs were partially reversed by the knockdown of GPR68 with siRNA [63] (see Figure 2). Therefore, GPR68 exerts regulatory functions with regard to the proliferation, differentiation, and migration of VECs and VSMCs within an acidic microenvironment. Further investigation is required to elucidate the complete role and mechanism of GPR68 in regulating arterial function at different pH values.

### 3.2. GPR4

The GPR4 gene is located on chromosome 19q13.3 and encodes a protein consisting of 362 amino acids. It is widely expressed in various tissues, including the vascular system, lung, liver, and intestine [30]. GPR4 is a proton-sensing receptor that exhibits low activity at a plasma pH of 7.4. However, it can be fully activated in acidic conditions, which is crucial for regulating the proliferation, migration, and angiogenesis of VECs [64]. Additionally, it has been demonstrated that GPR4 facilitates EPC-induced angiogenesis by activating the signal transducer and activator of transcription 3 (STAT3)/vascular endothelial growth factor A (VEGFA) pathway in patients with coronary artery disease [65]. In cellular experiments, the modulation of Notch receptor 1 (Notch1) expression through the use of siRNA or Notch receptor inhibitors has been demonstrated to significantly enhance GPR4-induced endothelial vasculogenesis and lymphocyte transendothelial migration in human microvessels [66]. Furthermore, GPR4 has been shown to promote angiogenesis and maintain vascular integrity and stability by increasing the expression of VEGF receptors [67]. Activation of GPR4 in the acidic microenvironment of tumors has been shown to lead to the activation of the G12/13/Rho guanosine triphosphatase (GTPase) signaling pathway, which in turn results in the formation of paracellular gaps in VECs. This process is positively correlated with the expression of VEC proliferation markers [68].

Notably, Wenzel et al. [69] discovered that GPR4 activates the Gαq/11-related pathway in VECs within a low-pH microenvironment, resulting in impaired cerebrovascular reactivity and anxiety induction. Furthermore, GPR4 activates the C/EBP homologous protein (CHOP) pathway, which ultimately results in apoptosis in HK-2 cells and human umbilical vein endothelial cells (HUVECs) [70]. With regard to the phenomenon of inflammation, a low-pH microenvironment activates the GPR4 in VECs. This activation results in the expression of stress response genes related to the endoplasmic reticulum, including CHOP, activate transcription factor 3 (ATF3), and vesicle NOD-, LRR-, and pyrin domain-containing protein 3 (NLRP3) expression. Consequently, this leads to the onset of inflammatory responses and apoptosis [71]. In a mouse model of acute hindlimb ischemia-reperfusion, GPR4 has been demonstrated to mediate a number of key processes, including tissue edema, inflammatory exudate formation, endothelial adhesion molecule expression, and leukocyte infiltration. Specific knockdown or pharmacological inhibition of GPR4 has been observed to result in attenuated tissue inflammation [26]. In an acidic environment, GPR4 plays a role in the transcription of genes associated with inflammatory mediators (such as NF-κB, NF-κB1, and NF-κB2) and adhesion molecules (such as E-selectin, vascular cell adhesion molecule-1 (VCAM-1), and intercellular adhesion molecule-1 (ICAM-1)). The inflammatory response induced by acidosis is significantly reduced by the administration of a GPR4 antagonist [72] (see Figure 2). To sum up, the activation of GPR4 in a low-pH microenvironment appears to play a role in arterial inflammation and may contribute to processes such as apoptosis and atherosclerosis. Furthermore, while the majority of current research on GPR4 is focused on VECs and the regulation of VSMCs remains uncertain, it may still have a positive impact on arterial function by regulating VEC proliferation, migration, and angiogenesis in a low-pH microenvironment. It is imperative to acknowledge the dual effect of a low pH microenvironment on the regulation of arterial function via GPR4.

### 3.3. GPR132/G2A

GPR132, also known as G2A, has been demonstrated to induce cell cycle arrest in the G2/M phase, delay mitotic progression, and diminish the transforming potential of BCR-ABL. It was regarded as a cell cycle regulator that inhibits cell proliferation [41]. In contrast to other members of the G-protein family, GPR132 displays only minimal proton sensitivity [73]. There is currently a debate among the scientific community regarding the role of GPR132 in the control of inflammatory processes. Wu et al. [74] observed a significant elevation in the levels of the proton-sensing receptor GPR132 and the inflammatory factor tumor necrosis factor-α (TNF-α) in the peripheral blood cells of patients diagnosed with pulmonary hypertension, in comparison to other G proteins. GPR132 has been demonstrated to exacerbate the inflammatory response by stimulating the secretion of pro-inflammatory factors, specifically IL-6 and IL-8, and promoting calcium mobilization [75]. Moreover, it has been demonstrated that this protein can induce M1-type macrophage polarization at sites of inflammation [76]. However, alternative research has indicated that GPR132 may play a role in dampening inflammatory and autoimmune responses by influencing the migration of monocytes and macrophages [77].

Furthermore, in the acidic tumor microenvironment, activated GPR132 has the capacity to promote M2-like macrophage polarization and inhibit inflammatory processes [78]. In relation to arterial stiffness, Parks et al. [79] discovered through experimentation that elevated serum levels of high-density lipoprotein (HDL) had no effect on the initial stages of atherosclerosis in GPR132-deficient LDLR KO mice. In contrast, Bolick et al. and Johnson et al. [27,80] previously conducted research on GPR132-deficient ApoE KO mice as an alternative animal model. It was observed that during the mid to late stages of atherosclerosis, there was an increase in the number of monocytes and pro-inflammatory M1-type macrophages. This influx was accompanied by enhanced adhesion of VECs, increased apoptosis, and larger atheromatous plaques. It may therefore be the case that GPR132 exerts a time-dependent influence on the development of atherosclerosis, with a reduced impact in the early stages of the disease and a manifesting of its pro-lesion properties during the intermediate and advanced stages of atherosclerosis (see Figure 2). In conclusion, while GPR132 may have a bidirectional function, primarily in macrophage-mediated pro-inflammatory and anti-inflammatory responses, there is currently a paucity of studies examining the proton sensitivity of GPR132. Additionally, the precise molecular mechanisms underlying the physiological functions of monocytes and macrophages in the regulation of atherosclerosis remain to be elucidated.

### 3.4. GPR65/TDAG8

GPR65, also known as TDAG8, was initially identified by Ishii et al. [81] as a proton-sensing receptor. It is located on chromosome 14q31-32.1 and exhibits a distinctive expression profile within the immune system. TDAG8 is predominantly expressed in cells of the immune system [82]. In humans, TDAG8 is predominantly expressed in peripheral blood leukocytes and lymphoid tissues, including the spleen, lymph nodes, and thymus [83], which suggests a pivotal role in innate and adaptive immune responses. Moreover, it is implicated in the accumulation of cAMP, Rho activation, and stress fiber formation. In response to extracellular acidification, TDAG8 plays a pivotal role in leukocyte migration and phagocytosis through the activation of the Gαs/AC/cAMP/PKA pathway, the G12/13 proteins, and the Rho-related pathway [81,84]. With respect to the arterial system, the primary function of this protein is to regulate the process of atherosclerosis.

In ApoE KO mice, an elevation in mRNA and protein expression levels of GPR65 was observed. Immunofluorescent staining has shown that GPR65 is predominantly distributed within proliferating cell nuclear antigen (PCNA)-positive VSMCs. It is proposed that this activation may regulate the phenotypic transformation, proliferation, and migration of VSMCs by activating the downstream cAMP/PKA pathway. This cascade subsequently exerts an influence on the pathological process of atherosclerosis [28]. It is proposed that GPR65 may serve as a pivotal regulator and therapeutic target in the context of myocardial infarction (MI). In the aftermath of MI, GPR65 prompts an uptick in lactate generated by myocardial anaerobic glycolytic metabolism and myocardial immune cell infiltration, culminating in cardiac ischemic acidosis. Mice lacking the GPR65 gene have been observed to overproduce IL-17A, which contributes to the deterioration of cardiac function following MI, resulting in a significantly lower survival rate and cardiac function compared to wild-type mice [85] (see Figure 2). Taken together, GPR65 contributes to the regulation of arterial function by mediating the proliferation, differentiation, and migration of VSMCs. A number of studies have investigated the role of GPR65 in arterial function, with a particular focus on its effects on plasma pH. The findings of these studies have consistently indicated that GPR65 has a beneficial effect on arterial function. Nevertheless, the precise mechanism by which GPR65 functions as a proton-sensing receptor in arterial function remains to be elucidated.

## 4. The Effects of Exercise on the Regulation of Arterial Function

Modifications in blood flow and shear stress are known to precipitate arterial remodeling, which is contingent upon the presence of a fully functional endothelium [86,87]. Additionally, VECs are instrumental in regulating vascular tone and blood pressure [88]. The hemodynamic changes that occur in response to exercise can directly or indirectly affect the diastole of arterioles, thereby prompting adaptive alterations in the arteries. These changes are mediated by the regulation of the function of both VECs and VSMCs [21,89,90,91]. The available evidence indicates that regular exercise may delay the onset of age-related arterial lesions. The impact of exercise on arterial function is subject to variation in terms of the type, intensity, and frequency of activity, and there may be heterogeneity in the effects on different age groups and populations. Research has shown that low- and moderate-intensity aerobic exercise can prevent the development of atherosclerosis in large arteries and improve endothelial function in sedentary middle-aged and older adults. Moreover, it has been demonstrated to reduce arterial stiffness and restore endothelial function [92]. It has been demonstrated that low-intensity exercise is significantly less effective than high-intensity exercise in improving exercise capacity in patients with peripheral arterial disease [93]. Additionally, research findings suggest that exercise training can reduce arterial stiffness and improve arterial compliance in the context of atherosclerosis [94,95]. Another study found that high-intensity resistance exercise resulted in a reduction in arterial compliance and an increase in arterial stiffness, whereas low-intensity resistance exercise led to a notable increase in arterial compliance and a decrease in arterial stiffness [96]. In summary, different types of exercise can induce modifications in arterial pressure, blood flow, and shear stress, which consequently lead to alterations in arterial diastolic function and stiffness (see Table 2).

In the field of research examining the impact of varying exercise durations on arterial function, acute aerobic exercise has been demonstrated to be effective in reducing central arterial stiffness, wave reflections, and hemodynamics in healthy populations [97,98,99]. Nevertheless, acute resistance exercise has been demonstrated to result in a transient increase in central artery stiffness [100]. Other studies have found that high-intensity aerobic exercise, or HIIT, three days per week for eight weeks has been found to be more beneficial in improving arterial stiffness in younger populations compared to acute aerobic or resistance training [101,102]. Long-term aerobic exercise (lasting for 12 weeks) has been shown to significantly improve atherosclerotic coronary endothelial dysfunction and reduce arterial inflammation and oxidative stress [103]. Another investigation demonstrated that long-term aerobic exercise (with an intervention period of 12–20 weeks) lessened arterial stiffness in hypertensive women [104]. A similar discovery was made in a year-long program of moderate-to-high-intensity aerobic exercise, which led to a significant decrease in carotid artery stiffness and enhancement in cerebral blood flow in patients with amnestic mild cognitive impairment [105]. Furthermore, a combination of long-term aerobic and resistance exercise (lasting between 6 and 12 months) has been demonstrated to significantly enhance arterial blood pressure and arterial function in patients with chronic kidney disease [106]. Conversely, an 8-week resistance exercise intervention had no effect on pulse wave velocity or arterial stiffness in patients with metabolic syndrome [107]. The current body of research suggests that the effect of exercise on arterial function is time-dependent. Specifically, arterial stiffness and compliance appear to be more positively impacted by prolonged exercise durations. However, it should be noted that the effects on arterial function are influenced by numerous exercise-related factors, including the intensity and nature of the exercise modality. Furthermore, the potential benefits in diverse disease states necessitate additional investigation.

## 5. Regulation of Arterial Function by Exercise Through Proton-Sensing GPCRs

It is established that both proton-sensing GPCRs and exercise play a significant role in the regulation of arterial function. Systemic acidosis, resulting from respiratory dysfunction (e.g., compromised pulmonary ventilation) or metabolic disorders (e.g., lactic acidosis), induces a moderate elevation of plasma H⁺ concentration. In contrast, localized acidic microenvironments observed in pathological contexts (e.g., ischemic tissues or solid tumors) are primarily driven by compartmentalized proton generation through accelerated glycolytic metabolism (Warburg effect) and dysfunctional ion transport mechanisms. This spatial heterogeneity disrupts physiological pH homeostasis, overwhelming endogenous buffering systems including bicarbonate (HCO_3_^−^/CO_2_) and protein-based buffers. In the absence of pathological conditions, exercise is the primary factor responsible for altered pulmonary ventilation and metabolism. This may result in a transient decrease in body pH. The objective of this section is to examine the potential mechanisms through which exercise influences arterial function via proton-sensing GPCRs.

### 5.1. The Effect of Exercise on the Generation of an Acidic Microenvironment

#### 5.1.1. Regulation of Acid–Base Balance During Respiration and Metabolic Stress

**Figure 3:**
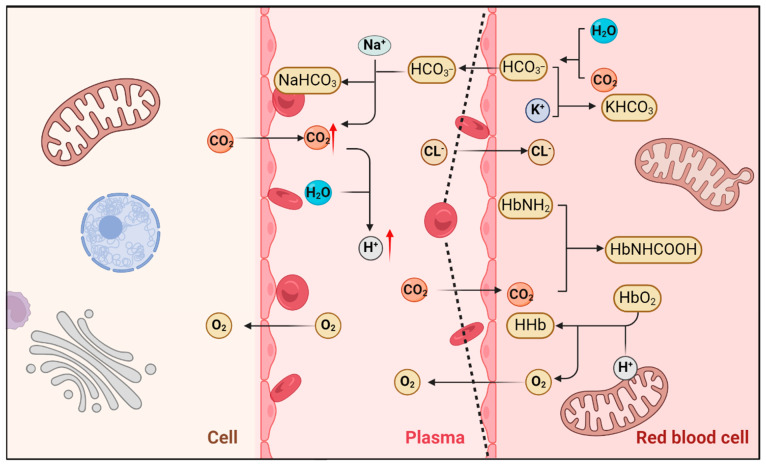
Regulation of acid–base balance during respiration and metabolic stress. During respiration, the lungs eliminate CO_2_ derived primarily from aerobic metabolism in muscle cells, as well as CO_2_ generated through the buffering of excess H⁺ by bicarbonate (HCO_3_⁻) in the blood. Concurrently, erythrocytes facilitate the transport of oxygen to tissue cells. When pulmonary ventilation or metabolic patterns are altered (e.g., during intense exercise or metabolic stress), the production of H⁺ may transiently exceed the capacity of pH buffer systems to neutralize it. This results in the accumulation of H⁺ in the plasma, leading to a decrease in arterial pH (e.g., from 7.4 to 7.2–7.3) and the development of a mild acidotic state. This figure was created using BioRender. Abbreviations: NaHCO_3_: sodium hydrogen carbonate; HHb: un-ionized hemoglobin; HbO_2_: oxyhemoglobin; HbNHCOOH: carbamino hemoglobin; HbNH_2_: aminohemoglobin; KHCO_3_: potassium bicarbonate; CO_2_: carbon dioxide; H^+^: hydrogen ion; H_2_O: water; HCO_3_^−^: bicarbonate ion.

The regulation of the plasma’s acidic environment is primarily maintained by buffer systems (e.g., the bicarbonate buffer system), respiratory control of CO_2_, and renal excretion of H⁺ and reabsorption of HCO_3_⁻. At the cellular level, intracellular pH and acid–base balance are regulated by membrane transport proteins, including the sodium–hydrogen antiporter 1 (NHE1), adenosine triphosphatase (ATPase), monocarboxylate transporters (MCT1 and MCT4), as well as Na⁺-HCO_3_⁻ cotransporters and Cl⁻/HCO_3_⁻ exchangers [108,109]. Pulmonary ventilation eliminates carbon dioxide (CO_2_) from venous blood via alveolar gas exchange. Under normal physiological conditions, CO_2_ produced by muscles during aerobic metabolism is efficiently removed by pulmonary ventilation. However, during lactic acidosis, the buffering of excess H⁺ by bicarbonate (HCO_3_⁻) generates additional CO_2_ through the reaction. This increases total CO_2_ production, potentially overwhelming the compensatory capacity of pulmonary ventilation. Consequently, dissolved CO_2_ and carbamino-bound CO_2_ levels rise, further lowering plasma pH [110,111]. Carbonic anhydrase (CA) catalyzes the hydration of CO_2_ to H⁺ and HCO_3_⁻, amplifying acidification. While the tricarboxylic acid (TCA) cycle is the primary source of cellular CO_2_, its overproduction during metabolic stress exacerbates acidosis [112]. During exercise, alterations in the body’s metabolic pattern lead to a decrease in arterial pH due to the limited capacity of pH buffers to regulate and remove the persistently increased levels of H^+^ in a timely manner [113,114]. Consequently, the regulation of plasma and intracellular acid–base homeostasis is dependent on a variety of mechanisms, including the buffering system, respiratory control, and renal regulation. However, during periods of strenuous exercise or metabolic stress (e.g., lactic acidosis), the overproduction of H^+^ and CO_2_ may temporarily exceed the body’s compensatory capacity, resulting in a decrease in arterial pH and an exacerbation of acidosis (see Figure 3).

#### 5.1.2. Exercise and the Regulation of Acid–Base Balance

**Figure 4:**
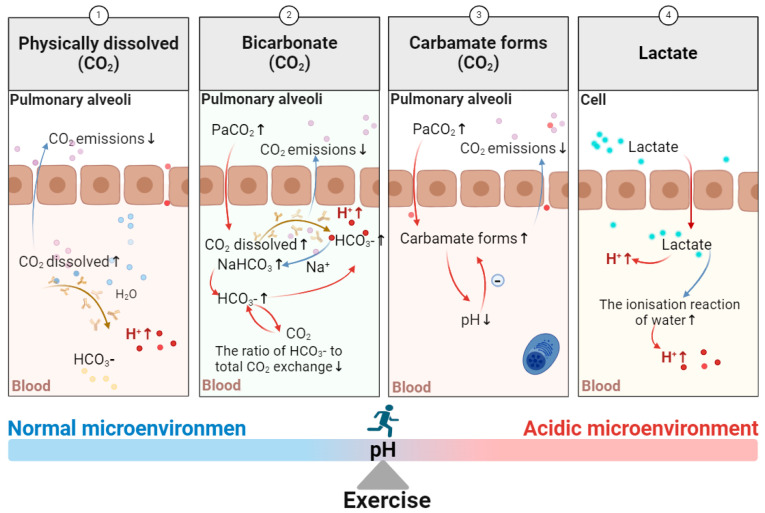
The effect of exercise on the formation of an acidic microenvironment. The metabolic processes occurring within muscle cells during exercise result in the production of CO_2_ and lactate. However, these byproducts cannot be eliminated from the body in an expedient manner, which leads to the formation of a weakly acidic microenvironment. This figure was created using BioRender. Abbreviations: PaCO_2_: carbon dioxide tension.

The duration of the isocapnic buffering period is contingent upon two variables: the quantity of available CO_2_ and the threshold of arterial carbon dioxide tension (PaCO_2_) necessary to stimulate ventilation via chemoreceptors [115]. During exercise, CO_2_ diffuses from the intracellular space into the convective transport medium, namely the blood, which comprises two compartments: plasma and erythrocytes. Subsequently, the CO_2_ enters the alveoli via the pulmonary capillary barrier and is eliminated from the body via pulmonary gas exchange. Carbon dioxide is transported in physically dissolved, bicarbonate, and carbamate forms [116]. During periods of rest, only 5% of CO_2_ is stored in its physically dissolved form within the arteries, representing a mere 10% of the total arteriovenous CO_2_ concentration difference. However, the proportion of CO_2_ physically dissolved increases to one-third of the total CO_2_ exchange during exercise [117]. During periods of strenuous exercise, the concentration of carbamates in arterial blood is higher than that in venous blood. Moreover, elevated CO_2_ partial pressures can precipitate an increase in venous blood carbamates, which can be offset by a reduction in carbamates due to a decline in pH [118]. The concentration of plasma HCO_3_^−^ levels is 13 times greater than that of physically dissolved CO_2_. During periods of intense physical exertion, the pH level of the plasma decreases to approximately 7.2, resulting in a 20-fold increase in the concentration of HCO_3_^−^ [119]. However, the relative contribution of HCO_3_^−^ to total CO_2_ exchange is diminished during exercise in comparison to rest. Specifically, HCO_3_^−^ accounts for only two-thirds of the total CO_2_ exchange during strenuous exercise, compared to 85% at rest (see Figure 4). Peronnet et al. [120] identified a robust correlation between alterations in arterial blood pH and pulmonary ventilation through empirical investigation. It has been postulated that from the initial ventilation threshold, VT1, to the maximum exercise intensity, the organism regulates plasma acid–base homeostasis by eliminating non-metabolic CO_2_ from the alkaline reserve.

Furthermore, exercise has been demonstrated to facilitate the accumulation of lactate. It was previously assumed that lactate was the exclusive product of anaerobic metabolism in skeletal muscle. However, recent evidence suggests that it can also be produced during the latter stages of aerobic exercise [121]. The energy molecule adenosine triphosphate (ATP) facilitates muscle contraction by enabling cross-bridge cycling between actin and myosin in the presence of myosin ATPase, which results in muscle stress. However, during sustained exercise, the ATP reserves of skeletal muscle are depleted, necessitating the reliance on phosphocreatine (PCr) and myoglycogen to facilitate the formation of pyruvate and ATP [122]. During strenuous exercise, this pathway is employed to produce ATP, thereby meeting the demands of the cross-bridge cycle and ion pump operation. This necessitates enhanced activity of the Ca^2+^-ATPase and Na^+^-K^+^-ATPase. However, during low-intensity exercise, the mitochondria are unable to fully oxidize the pyruvate produced, resulting in its accumulation in the sarcoplasm and conversion to lactate [123]. Recent studies have demonstrated that lactate can also function as a strong acid anion, promoting the ionization reaction of water and thus the production of H^+^ [124] (see Figure 4).

A study was conducted in which six male participants engaged in 30 min of resistance and isocaloric endurance exercise. The findings indicate that resistance exercise resulted in elevated lactate levels in comparison to endurance exercise, which in turn led to enhanced metabolic activity during the recovery period [125]. A retrospective analysis revealed that blood lactate concentrations in individuals with multiple sclerosis (MS) were comparable to those of healthy controls during acute sub-extreme and maximal-intensity exercise. However, it was observed that moderate-intensity exercise resulted in a notable reduction in blood lactate levels [126]. Lee et al. [127] conducted experiments demonstrating that both anaerobic and endurance exercise resulted in elevated levels of blood lactate and plasma H^+^. Furthermore, the administration of short-term creatine has been observed to mitigate the elevation in blood lactate concentrations that occurs in response to late-phase anaerobic or endurance exercise. The results of the Wingate test and incremental tests on cyclists with varying exercise levels and aerobic capacities indicate that both arterial capillary lactate and H^+^ concentrations were elevated three minutes after exercise. Additionally, mountain bikers demonstrated more pronounced anaerobic effects during incremental tests in comparison to road cyclists [128]. Both the moderate repetition protocol (MRP) and the high repetition protocol (HRP) resulted in an increase in blood lactate concentrations and a decrease in blood pH. The effect was more pronounced with HRP than with MRP. The elevation in blood lactate concentration and the decrease in blood pH were more pronounced with HRP than with MRP [129]. The administration of high-intensity interval training (HIIT) resulted in a notable elevation in blood lactate levels and a considerable augmentation in acidic ion pressure during the exercise period [130]. Prior research has indicated that lactate accumulation is directly linked to the production of H^+^, which causes a decrease in intramuscular pH or acidosis [131]. In conclusion, the data demonstrate a significant increase in blood lactate levels during both anaerobic and late aerobic exercise, which was accompanied by a subsequent rise in plasma H^+^ levels, and a corresponding reduction in the body’s circulating pH. The manner in which exercise affects circulating pH through lactate secretion is analogous to the effects of pulmonary ventilation. However, high-intensity exercise may result in a reduction in plasma pH, which is linked to unfavorable conditions such as metabolic acidosis within the body.

During low to moderate-intensity exercise, the body maintains a stable PaCO_2_ level due to balanced CO_2_ production and elimination through increased ventilation. In contrast, high-intensity exercise leads to the accumulation of lactic acid due to anaerobic metabolism, resulting in a reduction in plasma pH and the development of metabolic acidosis. The effects of exercise of differing intensities on the accumulation of protons within the organism are not uniform. In general terms, during moderate-intensity exercise, the body’s plasma proton concentration increases to a certain extent, yet it remains within a relatively stable and adjustable range. During this process, the body is able to make full use of oxygen, thus gradually oxidizing and breaking down carbohydrates, fats, and other nutrients. The rate of proton production is relatively smooth and slow, and aerobic metabolism has well-developed metabolic pathways and buffer systems to deal with the small number of protons produced by metabolism [132]. Intracellularly produced protons can be transported and neutralized by proton transport proteins (e.g., NHE1, etc.) in the cell membrane and buffering substances (e.g., HCO_3_^−^, etc.) in the bloodstream, thereby maintaining relatively stable intracellular and blood proton concentrations [108,109,110]. However, the rate and extent of proton production during high-intensity exercise far exceeds that of moderate-intensity exercise [133]. During periods of high-intensity exercise, there is a significant increase in the incidence of anaerobic metabolism within the body. The process of anaerobic glycolysis produces significant quantities of metabolites, including lactic acid, over a relatively brief period. Concurrently, this process gives rise to substantial H^+^ release [134,135]. Despite the body’s inherent proton transport and buffering mechanisms, it is unable to effectively respond to the rapid accumulation of large amounts of acidic protons over a short period of time. The intracellular concentration of protons increases rapidly, and there is a tendency for the blood to acidify [136]. In summary, the changes in the body’s proton concentration are relatively moderate during moderate-intensity exercise, whereas proton concentration increases rapidly and substantially during high-intensity exercise, and a longer time is required for subsequent recovery to normal levels.

In addition to exercise, other stressors such as hypoxia, hypertension, and the tumor microenvironment have been demonstrated to affect extracellular proton concentrations. It has been demonstrated that tissue hypoxia can induce glycolysis through the activation of the HIF-1α pathway. This, in turn, results in the production of substantial quantities of lactate and the consequent development of persistent acidosis [137]. In contrast to the phenomenon of exercise-induced acidosis, the condition of hypoxia-associated acidosis is characterized by an imbalance between oxygen supply and demand, accompanied by impaired ATP synthesis and oxidative stress [138]. Furthermore, in hypertension, the vascular endothelium releases protons (via ASIC1 channels) and inflammatory factors under high shear, leading to acidification of the local microenvironment [139,140]. In contrast to the physiological changes that occur in response to exercise, the process of acidification in hypertension represents a pathological alteration, characterized by endothelial dysfunction and vascular remodeling. It has been established that cancer cells within the tumor microenvironment maintain intracellular alkalinization and extracellular acidification by overexpressing CA IX/XII and ATPase, thus promoting invasion and drug resistance [141]. The reverse pH gradient observed within tumors is attributable to malignant metabolic reprogramming. In conclusion, when compared to persistent acidosis triggered by pathological factors such as hypoxia, hypertension, and the tumor microenvironment, the exercise-induced plasma pH change is indicative of enhanced metabolic vitality and physiological stress adaptation of the organism.

### 5.2. The Potential Mechanisms by Which Exercise Improves Arterial Function Through the Activation of Proton-Sensing GPCRs

**Figure 5:**
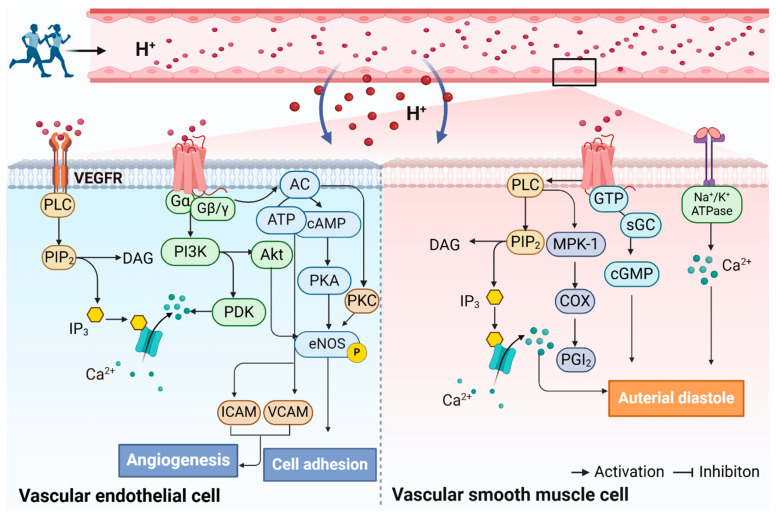
Possible mechanisms of exercise-mediated modulation of arterial function by the extracellular acidic microenvironment via proton-sensing GPCRs. Modifications in extracellular pH, occurring during and following exercise, have the potential to influence arterial functionality by regulating signal transduction in VECs and VSMCs. These changes are involved in regulating a number of processes, including apoptosis, adhesion, inflammation, arteriolar diastole, and angiogenesis. This figure was created using BioRender. Abbreviations: GTP: guanosine triphosphate; IP3: inositol triphosphate; DAG: diglyceride; sGC: soluble guanylyl cyclase; cGMP: cyclic guanosine monophosphate; MPK-1: mitogen-activated protein kinase phosphatase-1; COX: cyclooxygenase; PGI2: prostaglandin-I-2; Na^+^/K^+^-ATPase: sodium-potassium ATPase; PLC: phospholipase C; PIP2: guanosine triphosphatase; IP3: inositol triphosphate; DAG: diglyceride; PDK: phosphoinositide-dependent protein kinase; Calm: calmodulin; PI3K: phosphatidylinositol 3-hydroxy kinase; Akt: protein kinase B; ATP: adenosine triphosphate; AMPK: AMP-activated protein kinase; AC: adenylate cyclase; cAMP: cyclic adenosine monophosphate; PKA: protein kinase A; VCAM-1: vascular cell adhesion molecule-1; ICAM-1: intercellular adhesion molecule-1.

The regulation of plasma pH during exercise is contingent upon the equilibrium between CO_2_ production, irrespective of whether the activity in question is aerobic or anaerobic. The typical range for arterial blood H^+^ concentrations is 38–40 nmol/L, which corresponds to a pH of approximately 7.42 in a resting state [142]. During exercise, the accumulation of H⁺ in arterial blood primarily results from anaerobic metabolism and lactic acid production. The subsequent increase in ventilation (VE) serves as a compensatory mechanism to mitigate this acidosis by enhancing CO_2_ excretion, thereby preventing a sustained elevation of arterial H⁺ concentration [143]. The accumulation of plasma H^+^ following exercise activates proton-sensing GPCRs on cell membranes, particularly VECs and VSMCs. These receptors regulate a number of cellular processes, including proliferation, differentiation, migration, apoptosis, inflammation, and the growth of new blood vessels. Furthermore, they may play a pivotal role in regulating arterial function (see Figure 5).

The extracellular acidic microenvironment has been demonstrated to exert an influence on the activity and function of both VECs and VSMCs [144]. The extant research evidence is exclusively in vitro cellular experimentation. In a study, acid-pretreated ECs were found to prevent autoapoptosis by promoting p38 MAPK and Akt-dependent B-cell lymphoma extra large (Bcl-xL) overexpression [145]. Dong et al. [146] identified that the cAMP/exchange protein activated by 3′-5′-cyclic adenosine monophosphate (Epac) pathway is activated in HUVECs during acidosis, leading to the secretion of VCAM-1 and ICAM-1. The activation of the cAMP/Epac pathway in HUVECs facilitates the secretion of VCAM-1 and ICAM-1. Additionally, it induces the expression of numerous pro-inflammatory genes, including chemokines, cytokines, adhesion molecules, genes associated with the NF-κB pathway, COX-2, and stress-responsive genes. In a study by Namkoong et al. [147], it was demonstrated that forskolin, a cAMP agonist, stimulates angiogenesis by inducing PKA-dependent VEGF expression and Epac-mediated synergistic effects on the downstream PI3K/Akt/endothelial nitric oxide synthase (eNOs) pathway. It has been demonstrated that the acidity of the microenvironment regulates apoptosis, adhesion, and angiogenesis in VECs. Conversely, in VSMCs, an extracellular acidic microenvironment has been observed to increase the expression of MAPK phosphatase 1 (MKP-1), a phosphatase of ERK and p38MAPK, and LPA accumulation in the region of atherosclerotic lesions. This accumulation acted synergistically to promote GPR68-mediated COX-2 and MKP-1 expression [62]. Meanwhile, Tomura et al. [61] discovered that the activation of the OGR1/PLC/Ca^2+^/extracellular signal-regulated kinase (ERK)/COX/ PGI2 signaling pathway, through the use of siRNAs and specific inhibitor intervention methods, results in an increase in cAMP accumulation and PGI2 production, while simultaneously inhibiting the development of atherogenesis. Furthermore, acidosis has been demonstrated to induce vasodilation by activating eNOS and ATP-sensing potassium channels in the systemic vasculature. This, in turn, results in the hyperpolarization of VSMCs [40,148]. The acidic microenvironment in plasma exerts a considerable influence on vascular inflammation, diastole, and generation by modulating intracellular downstream signaling pathways in VECs and VSMCs (see Figure 5).

## 6. Conclusions

One of the principal factors responsible for fluctuations in the pH of an organism’s microenvironment is physical exercise. During exercise, alterations in the concentration of carbon dioxide and lactate may result in an increase in the concentration of hydrogen ions in the plasma. Research has indicated that proton-sensing GPCRs expressed on VECs and VSMCs have a significant impact on various arterial functions, including the proliferation, differentiation, migration, apoptosis, and angiogenesis of VECs and VSMCs. Furthermore, these receptors play a role in regulating the expression of inflammatory factors associated with atherosclerotic inflammation, including NF-κB, VCAM-1, ICAM-1, and TNF-α. The majority of previous research into proton-sensing GPCRs has predominantly concentrated on tumors and the perception of alterations in intracellular pH in tumor cells. This review is the inaugural investigation into the regulatory function of activated proton-sensing GPCRs in arterial function and the mechanisms by which they operate within the context of an exercise-mediated plasma acidic microenvironment.

The regulation of arterial function by proton-sensing GPCRs represents a developing area of research that requires further investigation to address certain outstanding issues. Despite the well-established regulation of arterial function by proton-sensing GPCRs, the precise direction and molecular mechanisms regulated by different receptors at varying pH levels remain unclear. This encompasses the specific effects of diverse signaling pathways and the cumulative influence of multiple receptors. The regulatory mechanisms of proton-sensing GPCRs by different intensities of exercise have yet to be elucidated. Nevertheless, the evidence indicates that low- and medium-intensity exercise has a beneficial effect on arterial function, whereas high-intensity resistance exercise may result in augmented arterial stiffness. The impact of low circulating pH on arterial function following high-intensity exercise remains unclear. The currently available evidence pertains to the regulation of plasma pH by exercise and the influence of plasma pH on arterial function via proton-sensing GPCRs. Despite the existence of stratified studies, there is currently no experimental investigation that has evaluated whether exercise affects arterial function through direct modification of plasma pH via proton-sensing GPCRs.

The emerging role of proton-sensing GPCRs as pH-dependent regulators of arterial homeostasis establishes a novel paradigm for understanding exercise-mediated cardiovascular adaptation. Further research is required to elucidate the precise mechanism through which the exercise microenvironment regulates arterial function by activating proton-sensing GPCRs. This would provide a scientific basis for understanding the role of exercise in preventing arterial dysfunction at the molecular level. In turn, this would provide a reliable foundation for the development of medications and exercise prescriptions to treat arterial stiffness. Furthermore, disparities have been observed in the efficiency of the intracellular acid buffering system and proton transport mechanisms across various age groups. Intrinsic health variations and genetic distinctions have also been demonstrated to influence the number and activity of intracellular proton transport proteins, as well as the structure and function of proton-sensing GPCRs. From a sports medicine perspective, pharmaceuticals developed to regulate the activity of proton-sensing GPCRs in patients with arterial dysfunction may have different effects in patients with different genetic backgrounds, health levels, and ages. Consequently, the potential for disparities in exercise-induced alterations in proton concentration and proton-sensing GPCR activity based on individual variation in genetics, health levels, and ages merits further investigation.

**Table 1:** Signaling and pharmacological reagents of proton-sensing GPCRs.

Receptor	Location	Signaling	Protons	Agonist (EC50)	Antagonist (IC50)	Ref.
GPR68 (OGR1)	Human:14q32.11 NM_003485 Mouse: Chr 12 NM_175493	Gαq; Gαs; Gαi; G12/13	pH 7.8–5.6, maximum activity at pH 6.8	CARTPT (3.2 µM) MOsteocrin (0.4 µM); MCorticotropin (1.8 µM); 3,5-disubstituted isoxazoles (µM range); CART (1 µM); Pro-opiomelanocortin-derived peptide (1.3 µM)	Cu^2+^ (µM range); Zn^2+^ (µM range)	[44,149,150]
GPR4	Human: 19q13.32 NM_005282 Mouse: Chr 7 NM_175668	Gαs; Gαq; Gαi; G12/13	pH 7.6–5.6	ND	Compound 3b (67 nM); NE 52-QQ 57 (70 nM); NE 52-QQ57 (70 nM); Compound 39c (110 nM)	[44,141,142,143,144,145,146,147,148,149,150,151,152,153,154]
GPR132 (G2A)	Human:14q32.33 NM_001278695.2 Mouse: Chr 12 NM_019925	Gαs; Gαq	pH 8.2–6.6	9S-HODE (~0.5 µM); 11-HETE (~1 µM); N -palmitoylglycine (~800 nM); N-linoleoylglycine (~800 nM); ONC212 (~400 nM); 11,12-EET (~10 µM); 9,10-EpOME (~10 µM)	Lysophosphatidylcholine (~10 µM); Telmisartan (~10 µM); GSK1820795A (~1 µM)	[155,156,157,158,159]
GPR65 (TDAG8)	Human: 14q31.3 NM_003608 Mouse: Chr 12 NM_ 008152.3	Gαs	pH 7.2–5.7	Psychosine (3.4 µM); BTB09089 (active concentration > 5 µM)	ND	[81,160,161,162]

Note: ND, not determined.

**Table 2:** Regulation of arterial function by exercise.

Subjects			Exercise Intervention Program			Mode of Action	Ref.
Model	Characteristics	Sample Size	Type	Intensity	Duration		
C57BL/6, WT mice; ApoEtm1Unc, ApoE KO mice	5–6 weeks old	ND	Treadmill running	15 m/min at a 5° grade (60–80% of VO_2max_)	5 days/week; 60 min/session; 15–16 weeks	Endothelial dysfunction ↑	[103]
Patients with amnestic MCI	29 in SAT/19 in AET	70	Moderate-to-vigorous AET or stretching and toning (SAT)	Moderate to vigorous	12 months	VO_2peak_ ↑; carotid β-stiffness index ↑; CBF pulsatility ↑	[105]
Older sedentary overweight and obese men	67 ± 2 years, BMI: 30.3 ± 2.8 kg/m^2^	17 males	Progressive, aerobic exercise	70% maximal power	3 days/week; 50 min/session; 8 weeks	Endothelial function ↑, retinal arteriolar width ↑, cardiovascular risk ↓	[163]
Recreational athletes	45.9 ± 9.6 years	46 females/5 males	Endurance exercise	ND	ND	Coronary artery calcification →	[164]
Young men	Tealthy and recreationally active; 23 ± 2 years)	10 males	Incremental leg cycling exercise	50, 100, 150 Watts	30 min	Radial artery mean and anterograde SR ↑	[165]
Overweight men	21–30 years; BMI: 30 ± 3 kg/m^2^	8 males	Swimming training	50–80% HRmax	3 days/week; 55 min/session; 8 weeks	Carotid arterial stiffness, systolic blood pressure, Peripheral resistance ↓; blood flow velocity, flow rate, maximal, mean wall shear stress ↑	[166]
Healthy adults	22 ± 2 years; BMI: 22 ± 2 kg/m^2^	12	Cycling	85 ± 5% HRmax	30 min	ICA conductance ↑; vasodilation of the ICA ↑	[167]
Healthy adults	23 ± 4 years	11 females/9 males	Isometric handgrip training	30% of maximal voluntary contraction	3 days/week; four, 2 min unilateral contractions; 8 weeks	Endothelium-dependent vasodilation ↑	[168]
Healthy adults	61.0 ± 1.3 years	60	Aerobic exercise	Medium–high intensity	8 weeks	Arterial stiffness ↓	[169]
Overweight and obese adults	BMI: 30.5 ± 7.2	30	Aerobic exercise	ND	8 weeks	Arterial dysfunction ↓	[170]
Healthy adults	61 ± 2 years	4 females/7 males	Endurance exercise	70% VO_2max_	60 min/session; 10 days	FMD ↑; CAC ↑	[171]
Healthy adults	66 ± 1 years	5 females/6 males	Recumbent cycling	75–80% HRmax	30 min	Brachial artery FMD ↑	[172]
Healthy adults	YA: 26 ± 5 years; 23.8 ± 3.3 kg/m^2^ OA: 60 ± 6 years; 30.0 ± 5.5 kg/m^2^	21 young adults; 25 older adults	Unilateral maximal isokinetic knee flexion/extension exercise	1RM	3 sets; 10 reps	CCA strain time ↓	[173]
Patients with metabolic syndrome	51 ± 12 years	57	Endurance exercise	60–85% 1RM	8 weeks	cfPWV ↓; artery stiffness →	[107]
Healthy adults	18–30 years	14 females/12 males	Resistance exercise	75% 1RM	3 sets; 10 reps	Arterial stiffness↑	[174]
Healthy adults	24 ± 1 year	7 males	Eccentric exercise	High intensity	1 set; 50 reps	Carotid arterial compliance ↓; endothelial function ↓; β-stiffness index ↑	[175]
Sprague–Dawley rats	10 weeks old	40 males	Treadmill running; HIIT	30 m/min; High intensity	5 days/week, 60 min/ session; 8 weeks; 4 days/week, 8 weeks, 14 repeats of 20 s/session, 10 s pause between sessions	PWV ↑; central arterial stiffness ↓	[176]
Patients with CAD	71.8 ± 10.2 years	18	Endurance training; HIIT	60%; 85–90% HRmax	30 min; 10 interval training periods	Acute endurance training; AS ↓; resistance training AS ↑	[177]
Healthy adults	56 ± 5 years	25 females	Endurance exercise; resistance exercise	Medium to high strength	150 min/weeks endurance exercise; 2 or more days/weeks strength-based exercise	PCS ↑; PSR ↑	[178]
Patients with peripheral artery disease	50–80 years	12	Walking exercise; resistance exercise; combined exercise	ND	10 bouts of 2 min walking; 2 sets of 10 reps in 8 resistance exercises; 1 set of 10 reps in 8 resistance exercises + 5 bouts of 2 min walking	Artery stiffness ↓	[179]
Same-sex twins	31 monozygotic, 14 dizygotic pairs; 25.8 ± 6.0 years	90	Endurance exercise; resistance exercise	ND	3 months	FMD ↑; vascular function ↑	[180]
Patients with chronic kidney disease	55 years and older; CKD stages 3b–4	99	Endurance exercise; resistance exercise	40–70% HRmax	6 days/week, 90 min/session; 12 months	Arterial function ↑	[106]
Patients with bariatric surgery	8–45 years	40 females	Endurance exercise; resistance exercise	Moderate intensity; 50–75% 1RM	3 days/week; 60 min/session; 16 months	Arterial stiffness ↓	[181]
Sedentary older adults	64 ± 1 years	64	MICT; HIIT	70% HRmax; 4 × 4 min at 90% HRmax interspersed with 3 × 3 min active recovery at 70% HRmax	4 days/week; 8 weeks	MICT: carotid artery compliance ↑; cfPWV ↑ HIIT: carotid artery compliance →; cfPWV →	[182]
Healthy adults	23.5 ± 1.2 years	10	MICT; HIIT	40% HRmax; 85% HRmax	MICT: 40 min; HIIT: 1 min/session; 2 min between sets; total 26 min	Artery blood flow velocity →	[183]
Healthy adults	21.4 ± 0.8 years; 1.73 ± 0.03 m; 62.1 ± 6.4 kg	11 males	Interval training; interval exercise of semi-recumbent cycling	57.6 kJ/exercise session	12 min	ICA SR↑	[184]

Note: “↑”: increase; “↓”: decrease; “→”: no significant change; HR_max_: maximal heart rate; VO_2max_: maximal oxygen uptake; 1RM: maximal force for 1 repetition; ND, not determined; VO_2peak_: peak oxygen uptake; CBF: cerebral blood flow; ICA: internal carotid artery; SR: shear rate; L-FMC: low-flow mediated constriction; FMD: low mediated dilatation; PWV: pulse wave velocity; BMI: body mass index; CAD: coronary artery disease; AS: arterial stiffness; HIIT: high-intensity interval training; MICT: moderate-intensity continuous training; PWV: pulse wave velocity; FMD: flow-mediated dilation; CAC: circulating angiogenic cell; CCA: common carotid artery; PCS: peak circumferential strain; PSR: peak strain rate.

## Data Availability

Not applicable.

## Abbreviations

AC	adenylate cyclase
AMPK	AMP-activated protein kinase
AoSMCs	aortic smooth muscle cells
ASICs	acid-sensitive ion channels
ATF3	activating transcription factor 3
ATP	adenosine triphosphate
ATPase	adenosine triphosphatase
Bcl-xL	B-cell lymphoma extra large
CA	carbonic anhydrase
Calm	calmodulin
cAMP	cyclic adenosine monophosphate
CBX	carotid body excision
CHOP	C/EBP homologous protein
CO_2_	carbon dioxide
COX	cyclooxygenase
CT1-α1	collagen type 1-α1
CVDs	cardiovascular diseases
DAG	diacylglycerol
DDIT3	DNA damage-induced transcript 3
eNOs	endothelial nitric oxide synthase
Epac	exchange protein activated by 3′–5′-cyclic adenosine monophosphate
EPCs	endothelial progenitor cells
ERK	extracellular signal-regulated kinase
FICO_2_	fraction of inspired CO_2_
G2A	G2 accumulation protein
GRK	G protein-coupled receptor kinase
GTP	guanosine triphosphate
GTPase	guanosine triphosphatase
H^+^	hydrion
H_2_O	water molecule
HbNH_2_	aminohemoglobin
HbNHCOOH	carbamino hemoglobin
HbO_2_	oxyhemoglobin
HCO_3_^−^	bicarbonate radical
HDL	high-density lipoprotein
HHb	unionized hemoglobin
HIIT	high-intensity interval training
HRP	high repetition protocol
HUVECs	human umbilical vein endothelial cells
ICAM-1	intercellular adhesion molecule-1
IL-6	interleukin-6
IP_3_	inositol triphosphate
KHCO_3_	potassium bicarbonate
KIM-1	kidney injury molecule 1
KLF2	Kruppel-like factor 2
KO	knockout
MAPK	mitogen-activated protein kinase
MCAv	middle cerebral artery blood velocity
MCT1	monocarboxylate transporter
MI	myocardial infarction
MKP-1	MAPK phosphatase 1
MRP	moderate repetition protocol
MS	multiple sclerosis
Na^+^/K^+^-ATPase	Sodium–potassium ATPase
NAD^+^	nicotinamide adenine dinucleotide
NADH	reduced form of nicotinamide–adenine dinucleotide
NaHCO_3_	sodium hydrogen carbonate
NFKB1	nuclear factor kappa B subunit 1
NF-κB	nuclear factor k-binding
NHE1	sodium–hydrogen antiporter 1
NLRP3	NOD-, LRR-, and pyrin domain-containing protein 3
Notch1	notch receptor 1
OGR1	ovarian cancer G protein-coupled receptor
OSS	oscillatory shear stress
PaCO_2_	carbon dioxide tension
PCNA	proliferating cell nuclear antigen
PCr	phosphocreatine
PDK	phosphoinositide-dependent protein kinase
PGI_2_	prostaglandin-I-2
PI3K	phosphatidylinositol 3-hydroxy kinase
PIP_2_	guanosine triphosphatase
PKA	protein kinase A
PKC	protein kinase C
PLC	phospholipase C
proton-sensing GPCRs	proton-sensing G protein-coupled receptors
Ras	Rat sarcoma
RC	respiratory compensation threshold
RELB	RELB proto-oncogene, NF-KB subunit
Rho	Ras homologous
ROCK	Rho-associated protein kinase
sGC	soluble guanylyl cyclase
STAT3	signal transducer and activator of transcription 3
TDAG8	T-cell death-associated gene 8
TNF-α	tumor necrosis factor-α
VCAM-1	vascular cell adhesion molecule-1
VE	pulmonary ventilation
VECs	vascular endothelial cells
VEGFA	vascular endothelial growth factor A
VSMCs	vascular smooth muscle cells
7TMRs	7-transmembrane structural domain receptors
